# Effects of Hypoxia on Selected Psychophysiological Stress Responses of Military Aircrew

**DOI:** 10.1155/2021/6633851

**Published:** 2021-11-22

**Authors:** A. Bustamante-Sánchez, J. Gil-Cabrera, J. F. Tornero-Aguilera, Jesús Fernandez-Lucas, Domingo Jesús Ramos-Campo, V. J. Clemente-Suárez

**Affiliations:** ^1^Universidad Europea de Madrid, Faculty of Sport Sciences, Villaviciosa de Odón, Spain; ^2^Research Centre in Applied Combat (CESCA), Toledo, Spain; ^3^Applied Biotechnology Group, Universidad Europea de Madrid, Urbanización El Bosquen, E-28670 Villaviciosa de Odón (Madrid), Spain; ^4^Grupo de Investigación en Ciencias Naturales y Exactas, GICNEX, Universidad de la Costa, CUC, Calle 58 # 55-66. Barranquilla, Colombia; ^5^LFE Research Group, Department of Health and Human Performance, Faculty of Physical Activity and Sport Science-INEF: Universidad Politécnica de Madrid, Madrid, Spain; ^6^Grupo de Investigación en Cultura, Educación y Sociedad, Universidad de la Costa, Barranquilla 080002, Colombia

## Abstract

There is a lack of information on the psychophysiological response of pilots under hypoxic conditions. The study of the physiological, psychological, cardiorespiratory, neurological, behavioural, sensory, and cognitive symptoms that may appear during training in hypobaric chambers is essential to optimize the training processes of aircrew members. Thus, the present study is aimed at analyzing the psychophysiological responses of aircrew members in an incremental hypoxia training protocol. Psychophysiological responses of 44 aircrew members (34 males and 10 females) in an incremental hypoxia training protocol (3 minutes at 0 meters, 8 minutes at 5,000 meters, and maximum time at 7500 meters) were measured. Results suggested that the incremental hypoxia training protocol did not affect cortical arousal and handgrip strength; however, it increased the sympathetic tone, perceived stress, perceived effort, and heart rate and decreased forced expiratory volume and blood oxygen saturation. Thus, we concluded that acute hypoxic hypobaric exposure leads to decreased parasympathetic tone, blood oxygen saturation, and maximal spirometry values, without negatively affecting handgrip strength and cortical arousal. This information will lead to find specific training systems that meet the real needs of aircrew.

## 1. Introduction

Acute exposure to hypoxia has been recognized as one of the most serious single hazards during flight at altitude [1]. However, it is not the only risk factor for the aircrew. At higher speed and height and depending on the type of aircraft, the noises, vibrations, physical and mental fatigue, and spatial disorientation are highly variable [2], thus increasing the psychophysiological demands of the pilots and aircrew [3]. Therefore, evaluation of the effects of lower air pressure and increased altitude on the perceptual abilities and psychophysiological responses of aircrew and pilots has been widely studied since they are of practical and vital importance to optimize their responses. Thus, aircrew members receive extensive training using hypoxic chambers to simulate altitudes of 3,800 m and above to experiment and identify the physiological, psychological, cardiorespiratory, neurological, behavioural, sensory, and cognitive problems that may occur during hypoxia [4].

As the height increases, the air pressure decreases, leading to a lower oxygen partial pressure in the air. This lack of oxygen in the air causes a lower alveolar oxygen partial pressure in the lungs thus reducing oxygen partial pressure in the arterial blood. An acute ventilatory response is a mechanism that works to get back oxygen concentration homeostasis, causing hypocapnia and respiratory alkalosis [5], as well as causing breathing muscles fatigue [6]. The personal tolerance of low blood oxygen concentrations will trigger the different symptoms experienced, most of them caused by a lack of cerebral oxygen delivery. Although aviation pilots and aircrew have supplementary oxygen when flying above 3,800 m, however, in case of error or failure of oxygen delivery, blood oxygen saturation (SaO2) could decrease below 85% [4], leading to psychomotor, cognitive, and visual impairment along with increased stress and anxiety, shortness of breath, paraesthesia, headache, dizziness, nausea, light-headiness, and tachycardia [7–10]. Concentration difficulties, decrease in performance, defective judgment, and increase in both visual and auditory reaction times are also observed at the cognitive level [7], showing euphoria, overconfidence, lack of discipline, and irresponsibility [5]. Even short-term hypoxia exposure has shown negative effects on cognitive functions such as declarative memory and information processing, leading to inaccuracies on logic and mathematical tests [8] and acute memory loss and loss of cognitive performance [9].

Furthermore, hypoxia mainly affects the sensory receptors, the central nervous system (CNS), and the sympathovagal balance (autonomic tone) [5]. In this context, recent studies are focusing on the study of the critical flicker fusion threshold (CFFT) since it is a reliable, accessible, and noninvasive tool to assess the fatigue of the CNS [10]. Its impairment during hypoxia leads to compromised perceptual abilities of pilots [10]. In addition, the heart rate variability (HRV) analysis allows us to objectively determine the state of the autonomic tone [11]. However, its interpretation under hypoxic conditions shows ambiguities in the scientific literature. Some studies suggest sympathetic activation under hypoxic conditions of over 8,000 m, while others suggest that above 5,000 m, there is inhibition of the sympathetic tone and dominance of the parasympathetic one [12]. Yet, these discrepancies seem to be related to the nonlinear variable, spectrum, or time-domain variables used for the interpretation.

The lack of descriptive studies that delve into the effects of hypoxia exposure on the psychophysiological responses and cognitive functions has led to the present study. We aimed to analyze the psychophysiological responses of the aircrew members in an incremental hypoxia training protocol. According to previous research, we hypothesized that (i) hypoxia exposure would not decrease cortical arousal and parasympathetic tone of aircrew and (ii) blood oxygen saturation and strength index would decrease after hypoxia exposure.

## 2. Material and Methods

### 2.1. Participants

We analyzed 44 aircrew members (34 males and 10 females). The characteristics of the subjects are presented in Table 1.

All of them belonged to the Spanish Air Forces with a qualification of “fit” according to the periodic medical examination as recorded in the ministerial order 23/2011. The present research was carried out during the STANAG 3114 “Aeromedical Training of Flight Personnel” from NATO, approved by the ministerial order 23/2011 of the Ministry of Defence of Spain. Before starting the research, the experimental procedures were explained to all the participants, who gave their voluntary written informed consent in accordance with the Declaration of Helsinki. The procedures conducted in the present research were designed and approved by the Medical Service of the Aerospace Medicine Instruction Centre of Spanish Air Forces. All simulation was performed with the standard uniform boots equipment and flying operative helmet and mask.

### 2.2. Methods

#### 2.2.1. Procedure

We analyzed the psychophysiological responses of the participants in an incremental hypoxia training that followed this protocol: 3 minutes at 0 meters, 8 minutes at 5,000 meters, and maximum time they maintained (maximum of 10 minutes) at 7500 meters. Oxygen concentration and pressure were simulated according to the aforementioned heights. The different heights were used for a better understanding of hypoxia exposure at low (hypobaric hypoxia), middle, and high altitudes.

The training was supervised by the medical service and the training flight instructor of the Aerospace Medicine Instruction Centre. The end of the training could occur if one of these facts happened: medical service noticed health risk, participant noticed health risk, sudden heart rate rise detected by the training equipment, and blood oxygen saturation below 60% detected by the training equipment.

Before and immediately after the hypoxia exposure, the following parameters were analyzed:

*Bodyweight*: it is measured with a SECA scale model 714 with a precision of 100 grams (range 0.1-130 kg), located on a flat and smooth surface and calibrated at zero. Subjects were barefooted and with minimal clothes. Once located in the center of the platform, they remained without their body being in contact with surrounding objects, with the weight evenly distributed on both feet facing forward.

*Height*: it is measured with a SECA scale model 714 with a precision of 0.1 mm (range 60-200 cm). The subject stood up barefooted with the head oriented in the Frankfurt plane. Arms are on both sides of the trunk and fully extended with palms touching the external face of the thighs, heels together touching the lower end of the vertical surface with the inner edge of the foot, occipital area, scapular, buttocks, posterior face of the knees, and calves touching the vertical surface of the anthropometer.

*Subjective Scale of Distress (SUDS)*: it is ranging from zero (0), which implies “Completely indifferent and cold; does not affect me” to one hundred (100), which means “So distressed and tense that I can't deal with it”. This scale will be applied in the five minutes before the start of the session and provides information based on the level of stress assessed by the individual and which represents the cognitive relationship between the objective event and the emotional response.

*Rating of perceived exertion (RPE)*: it is 6 to 20 scale, scale through which the effort is identified numerically, with 6 being the minimum effort and 20 physical extenuations.

*Isometric handgrip strength by a TKK 5402 dynamometer (Takei Scientific Instruments Co. Ltd)*: each soldier's grip strength was measured for the dominant hand. Soldiers were placed sitting with 0 degrees of shoulder flexion, 90 degrees of elbow flexion, and the forearm in neutral. The average result of the two trials was recorded.

*Blood oxygen saturation by an oximeter OXYM4000 (Quirumed, Madrid)*: it is placed in the index finger of the right arm.

Spirometry values of forced vital capacity (FVC), forced expiratory volume in 1 second (FEV1), and peak expiratory flow (PEF) were measured using a QM-SP100 (Quirumed, Spain) spirometer, performing a maximum inhale-exhale-inhale cycle.

*Memory test*: a randomized number of 3 digits was presented for 1 second, and after 5 seconds, subjects had to inversely write it down, as in previous research [3].

Cortical arousal was measured through the critical flicker fusion threshold (CFFT). The subjects were seated in front of a viewing chamber (Lafayette Instrument Flicker Fusion Control Unit, Model 12021), which was constructed to control extraneous factors that might distort CFFT values. Two light-emitting diodes (58 cd/m^2^) were presented simultaneously in the viewing chamber, one for the left eye and one for the right eye. The stimuli were separated by 2.75 cm (center to center) with a stimulus-to-eye distance of 15 cm and a viewing angle of 1.9°. The inside of the viewing chamber is painted flat black to minimize reflection. The flicker frequency increment (2 Hz/sec) is from 20 to 100 Hz until the participant perceived fusion. After a fovea binocular fixation, participants were required to respond by pressing a button upon identifying the visual fusion thresholds. Before the experiment, they performed as many practice trials as needed to become familiar with the exigencies of the CFFT test. Then, five trials were performed, with an interval between them of 5 seconds. The final CFFT value was calculated as the average values of the five frequency tests. A decrease in this value indicates an increase in cortical arousal and information processing; on the contrary, an increase in the values would show central nervous system fatigue and a reduction in the efficiency of information processing systems [13].

Finally, heart rate (HR) and heart rate variability (HRV) were recorded before, during, and after the hypoxia training by a Polar V800 HR monitor (Kempele, Finland) with a sampling frequency of 1000 Hz, with which the RR intervals (time interval between R waves of the electrocardiogram) can be analyzed for the analysis of the HRV and the number of beats per minute for the HR analysis. Baseline, hypoxia, and recovery measures were taken in a prone position, while remaining in a waiting area, free of noises and distractions, in a controlled environment (23.1 ± 0.5°C temperature and 41.3 ± 2.0% of humidity) and at midday. Fifteen minutes was used as baseline time measure and 10 minutes as recovery; then, HRV was analyzed during the entire disorientation, as in previous research on military population [13]. Subsequently, the following parameters of the HRV time domain were analyzed using the Kubios HRV software program with no factor of correction, since the measures obtained were clean and free of noise (University of Kuopio, Kuopio, Finland).

*Time-domain (nonspectral) analysis*: this analysis was based on the assessment of the intervals between normal beats on 24-hour ECG recordings. During the statistical analysis, generally, all the QRS complexes, the duration between consecutive QRS complexes (NN interval), or the instantaneous heart rate during continuous ECG recordings are determined. We recorded the following time-domain indices: pNN50, which is the percentage of successive normal sinus RR intervals exceeding 50 ms (%) and RMSSD (ms) which is the square root of the mean value of the sum of squared differences of all successive RR intervals.

*Frequency-domain (spectral measures) analysis*: frequency-domain measures give information about how the power is distributed as a function of frequency. This analysis gives us smoother spectral components that can be distinguished as independent from preselected frequency bands and easy postprocessing of the spectrum with an automatic calculation of low frequency (LF) and high-frequency (HF) power components, easy identification of the central frequency of each component, and accurate estimation even on a small number of samples [13]. HF and LF (n.u) were measured in order to measure the peaks of parasympathetic, high-frequency component, frequency range: 0.15–0.40 Hz (HF), and sympathetic low-frequency component frequency range: 0.04–0.15 Hz (LF) values. In addition, LF/HF ratio was also evaluated.

*Nonlinear domain analysis*: SD1 and SD2 were measured to reflect the fluctuations of the HRV throw a Poincaré chart, physiologically, on the transverse axis. SD1 reflects parasympathetic activity while SD2 reflects the long-term changes of RR intervals and is considered as an inverse indicator of sympathetic activity.

### 2.3. Statistical Analysis

To analyze the data, we used the SPSS statistical package (version 24.0, SPSS. Inc. Chicago). Means and standard deviations (SDs) were calculated using traditional statistical techniques. Normality assumptions were checked with a Kolmogorov-Smirnov test. The differences between baseline and hypoxia samples were analyzed using a Student's *t*-test for paired samples. The differences in the three HRV moments analysis were calculated by an ANOVA of repeated measures. The effect size (ES) was tested by Cohen's D (ES = (hypoxia − test mean − baseline − test mean)/baseline − test SD). The level of significance for all the comparisons was set at *P* ≤ .05.

## 3. Results

The results are reported as mean ± SD.  Table 2shows the comparison of the physiological and psychological variables studied in aircrew personnel in the 2 moments: before and immediately after the finalization of the hypoxia training. A significant increase was found in the SUDS, RPE, FEV1, and SaO2 while a decrease in the FEV1 and SaO2 was observed due to hypoxia training.

In Table 3, the results of the autonomic modulation analysis in 3 moments, before the hypoxia, during the hypoxia, and after the hypoxia, are shown. Hypoxia exposure significantly decreased the RMSSD, LF, HF/LF ratio, SD1, and SD2, while a significant increase in the HR mean values was observed.

## 4. Discussion

This study is aimed at analyzing the psychophysiological responses of the aircrew members in an incremental hypoxia training protocol. The first hypothesis of the study is partially fulfilled since cortical arousal did not decrease, but parasympathetic tone decreased. The second hypothesis is also partially complied since blood oxygen saturation decreased but strength manifestation did not change.

cortical arousal has previously been studied in hypoxic [10, 14] and real flight conditions [15, 16] since it usually negatively affects cortex performance. It has been found to be mediated by hypercapnic stimuli since the central chemoreceptors touch off alveolar respiration through brainstem respiratory centers [11, 16]. In this line, cortical arousal was not expected to be affected by hypoxia exposure as previous researchers found no significant differences in a recent study with a higher sample of pilots and aircrews, highlighting the need to gather longer samples [17]. Nevertheless, in hypobaric chamber training, authors have reported a significant decrease in cortical arousal with the decrease of oxygen saturation in pilots [10]. Although this kind of training is difficult to analyze due to the availability of material and human resources involved, the results of the present research confirm that cortical arousal does not change as expected, maybe because of underlining mechanisms of anticipatory response that help to balance homeostasis in the expectancy of a hazard [14].

Regarding the SaO2, we found a significant decrease after the hypoxia exposure. This response was in line with previous researches in hypoxia conditions [10, 18] showing how hypoxia acts as a stressor to the participants, who reported a significant increase in perceived stress. A decrease in SaO2 usually predicts a significant increase in HR, which is a physiological adaptation to deliver the same amount of oxygen by augmenting the cardiac output [5]. The results obtained in the present research are similar to the previous hypoxia researches [18, 19]. Regarding breath muscle strength analysis, FEV1 decreased after hypoxia training. The need to maintain a similar oxygen uptake with less oxygen partial pressure had an impact on breath muscle fatigue after the hypoxic. These results coincided with previous researches, highlighting that PEF and FEV1 are the metrics primarily affected, which are related to explosive breath muscle strength in both hypoxic [14] and real flight conditions [3, 15].

Regarding muscle strength, different outcomes have been described under hypoxia that depends on the training and role of the pilot. For instance, a moderate effect size increase was observed for the transport pilots but no effects were observed for other types of pilots and aircrew personnel [14]. The results obtained in our study were in contrast to the initial hypothesis since no changes in isometric hand strength were found. This explains that muscle fibers' activity did not diminish in response to the hypoxia but rather shifted from a slow to faster oxidative profile, thus without affecting overall performance [3]. It could also be explained by a balance of both sympathetic activation after a risk is perceived and muscle fatigue due to a lack of oxygen [15], as well as the fact that in the evaluation test, the principal muscle fibers involved are fast-twitch fibers with anaerobic metabolism.

Heart rate variability has been traditionally used to assess autonomic tone (sympathetic or parasympathetic activation). With hypoxia-induced stress, HRV values tend to decrease, as previously reported by different researchers that mainly measured temporal and frequency domains [17, 19]. In the present research, RMSSD significantly decreased after hypoxia, which could be related to a dominance of the sympathetic tone that prepares the organism for a stressful situation. According to frequency domain analysis, there was a decrease in LF values [20], which could be interpreted as a decrease in sympathetic activation [21]. However, recent studies suggested a lack of such a relationship, since LF could be interpreted as sympathetic or/and parasympathetic outflow influenced by baroreflex modulation of cardiac autonomic outflow, vasomotor tone modulation, and mechanisms of central autonomic outflow. To date, there is no widely accepted interpretation of LF power; thus, most of the authors rely on time-domain values [21]. These results could be explained by a simultaneous decrease of sympathetic activation after the training and a progressive dominance of the parasympathetic tone to recover homeostasis. Since these mechanisms tend to occur gradually, future research should consider the prevalence of each during recovery periods.

LF/HF ratio, which is related to sympathetic activation, also increased during recovery, showing the duality of sympathetic/parasympathetic over the recovery period and indicating that this ratio is still controversial [22] and needs to be evaluated together with other metrics. Finally, nonlinear domain analysis (SD1 and SD2) showed a sympathetic activation during the hypoxia. This metric showed the dominance of the sympathetic system during hypoxia training (stressful situation) and parasympathetic activation during baseline (resting) and after hypoxia (recovery) periods. Since SD1 and SD2 metrics are preferred for short-term HRV measurements and they predict both linear and frequency domains [22–24], it may be further considered to analyze autonomic tone in hypoxia training. Finally, the significant increase of SUDS and RPE highlighted the large demands of hypoxic training [25].

### 4.1. Limitations of the Study

The main limitation of this study was the small sample size for each group and the difficulty to assess aircrew personnel, as they belong to an elite group among military forces. Secondly, there were no measurements of stress hormones (cortisol and adrenaline) and electroencephalography to better understand the psychophysiological response in hypoxia conditions of aircrew. Further research should be conducted to address hypoxia in both commercial and general aviation aircrews and in female pilots, especially.

### 4.2. Practical Applications

These results could help to design specific training to improve aircrews' preparation process facing hypoxic threats. Many of the recommendations still lack adequate specificity, and there is a need to take into account the real needs of flight personnel and their specific demands and characteristics. With a better understanding of the stress response that the aircrew personnel might suffer, it will be easier to find specific training systems that meet the real needs of aircrews.

## 5. Conclusion

Acute hypoxic hypobaric exposure produced a decrease in parasympathetic tone, blood oxygen saturation, and maximal spirometry values, without negatively affecting handgrip strength and cortical arousal in aircrew members.

## Figures and Tables

**Table 1:** Descriptive data of the subjects under study.

	Male aircrew	Female aircrew
Number of members	34	10
Age	37.9 ± 7.9	40 ± 3.3
Height (cm)	177.4 ± 6.8	163.2 ± 1.1
Weight (kg)	84.3 ± 9.8	64.4 ± 10.1
Years of military service	18.0 ± 9.4	17.5 ± 1.6
Months deployed	12.0 ± 12.2	8.0 ± 11.3
Flight hours	1253.5 ± 1695.2	2269.0 ± 2677.9.1

**Table 2:** The psychophysiological variables during the baseline and hypoxia.

							95% confidence interval	
Variables	Baseline	Hypoxia	*t* value	*P*	ES	% of change	Lower	Higher
SUDS (rank 0-100)	17.1 ± 22.1	40.4 ± 25.7	-7.667	≤0.001^∗^	1.05	136.2	-29.50	-17.2
RPE (rank 6-20)	7.7 ± 5.2	11.1 ± 2.6	-0.280	≤0.001^∗^	0.65	44.1	-5.01	-1.80
HS (kg)	47.7 ± 9.6	47.6 ± 10.1	0.078	0.939	-0.01	-0.2	-1.13	1.22
FVC (ml)	4.6 ± 0.9	4.4 ± 1.2	1.562	0.126	-0.22	-4.3	-0.59	0.46
FEV1 (ml)	3.7 ± 0.6	3.5 ± 0.8	2.063	.045^∗^	-0.33	-5.4	0.00	0.41
PEF (L/sec)	9.0 ± 2.9	8.6 ± 2.2	1.340	0.187	-0.14	-4.4	-0.21	1.04
SaO2 (%)	97.1 ± 1.4	74.0 ± 7.8	18.501	≤0.001^∗^	-16.5	-23.8	20.65	25.70
CFFT (Hz)	36.0 ± 3.7	36.8 ± 3.8	-0.762	0.450	0.22	2.2	-1.00	0.45

SUDS: Subjective Scale of Distress; RPE: rating of perceived exertion; HS: hand strength; FVC: forced vital capacity; FEV1: forced expiratory volume in one second; PEF: peak expiratory flow; SaO2: blood oxygen saturation; CFFT: critical flicker fusion threshold; ES: Cohen's D effect size; *t* value: Student's *t*-test.

**Table 3:** Heart rate variability.

	Evaluation moment				
Variables	Baseline	Hypoxia	Recovery	*P*	Moment comparison (1): baseline (2): hypoxia (3): recovery
HR mean (bpm)	77.8 ± 9.8	81.5 ± 12.6	76.5 ± 9.7	0.003^∗^	1 < 2; 2 > 3
HR max (bmp)	102.9 ± 13.9	102.4 ± 15.2	100.3 ± 9.9	0.375	
HR min (bmp)	61.7 ± 7.3	63.5 ± 9.4	61.7 ± 7.9	0.453	
RMSSD (ms)	38.5 ± 19.0	33.9 ± 14.7	37.7 ± 12.5	0.047^∗^	3 > 2
PNN50 (%)	17.8 ± 14.2	20.4 ± 20.7	20.6 ± 16.3	0.074	
LF (n.u)	71.0 ± 19.2	67.8 ± 18.7	75.4 ± 17.6	0.039^∗^	3 > 2
HF (n.u)	28.5 ± 18.7	30.6 ± 16.7	24.5 ± 17.5	0.064	
LF/HF (ratio)	7.2 ± 5.2	4.3 ± 2.7	8.1 ± 6.1	.005^∗^	1 > 2; 3 > 2
SD1 (ms)	23.4 ± 11.2	16.3 ± 5.3	23.4 ± 7.4	≤0.001^∗^	1 > 2; 3 > 2
SD2 (ms)	61.1 ± 19.8	45.4 ± 14.7	69.9 ± 17.6	≤0.001^∗^	1 > 2; 3 > 2

HR: heart rate; RMSSD: square root of the mean of the sum of the squared differences between adjacent normal RR intervals; pNN50: the percentage of differences between RR intervals higher than 50 ms; HF: high frequency; LF: low frequency; n.u.: normalised unit; SD1: variability of the short-term HRV; SD2: variability of the long-term HRV.

## Data Availability

All the data are included in the manuscript.
